# Targeting telomerase for cancer therapeutics

**DOI:** 10.1038/sj.bjc.6604209

**Published:** 2008-01-29

**Authors:** J W Shay, W N Keith

**Affiliations:** 1 Department of Cell Biology, University of Texas Southwestern Medical Center at Dallas, 5323 Harry Hines Boulevard, Dallas, TX 75390-9030, USA; 2 Centre for Oncology and Applied Pharmacology, University of Glasgow, Cancer Research UK Beatson Laboratories, Switchback Road, Bearsden, Glasgow G61 1BD, UK

**Keywords:** telomeres, targeted therapeutics, ageing

## Abstract

One of the hallmarks of advanced malignancies is continuous cell growth and this almost universally correlates with the reactivation of telomerase. Although there is still much we do not understand about the regulation of telomerase, it remains a very attractive and novel target for cancer therapeutics. Several clinical trials have been initiated, and in this review we highlight some of the most promising approaches and conclude by speculating on the role of telomerase in cancer stem cells.

Telomerase is a cellular reverse transcriptase (molecular motor) that adds new DNA onto the telomeres that are located at the ends of chromosomes (Collins and Mitchell, 2002). Telomeres consist of long TTAGGG nucleotide repeats and an associated protein complex, termed shelterin (de Lange, 2005). The shelterin complex protects chromosome ends from end-to-end fusion and degradation forming special t-loop like structures and thus masking the linear ends of chromosome from being recognised as single and/or double-strand DNA breaks. The TTAGGG repeats shorten with each cell division due to the end replication problem, oxidative damage and other end processing events (Harley, 1991; Wright and Shay, 2000). When a few telomeres become critically shortened there is a growth arrest state, at which time a DNA damage signal and cellular senescence are normally triggered (Shay, 2003). In the absence of other changes, cells can remain in a quiescent/senescent state for years and this can be thought of as a potent anticancer protection mechanism for long-lived species such as humans. However, human tumour cells derived from carcinomas almost universally bypass cellular senescence and DNA damage-signalling pathways. In cell line models, senescence bypass can be accomplished by abrogating important cell-cycle checkpoint genes (such as p53, p21, p16^INK4a^ and pRb), leading to extended growth of the premalignant cells eventually leading to crisis (Wright *et al*, 1989). Crisis is a period where cell growth and death are in balance. We believe that due to chromosome end fusions, there are chromosome breakage-fusion-bridge events, leading to genomic instability, rearrangements of chromosomes and eventually activation or upregulation of telomerase. Telomerase is detected in approximately 90% of all malignant tumours (Kim *et al*, 1994; Shay and Bacchetti, 1997), making it a highly attractive target for the development of mechanism-based therapeutics (Keith *et al*, 2007). The general schemes for targeting telomerase are well described (Keith *et al*, 2004, 2007; Shay and Wright, 2006). This review will therefore focus on discussing the most translational approaches, which are in the final stages of preclinical development and those that are already in clinical trials.

## OVERVIEW OF APPROACHES TO TELOMERASE THERAPEUTICS

Many investigators believe that targeting telomerase is a novel approach to targeted cancer therapeutics. This is not without some concerns, but in some instances therapy directed at telomerase has advanced to clinical trials to validate safety, to obtain maximum tolerable dose and in some cases to determine target specificity (Keith and Bilsland, 2008). Although there are many potential ways to interfere with normal telomerase function, only a few of the most promising approaches in preclinical and clinical trials will be described. Each approach has its own strengths and weaknesses (see Table 1), many of which will only be resolved through clinical trials involving biomarkers and pharmacodynamic endpoints. Only through the inclusion of pharmacodynamic endpoints will target specificity for telomerase be truly evaluated, and it is this last point which will be the most challenging for trial development (Keith *et al*, 2007).

There are three general classes of agents that have been developed to target telomerase biology; gene therapy; immunotherapy and small-molecule inhibitors (Keith and Bilsland, 2008). In this review, we will consider some approaches that are about to enter or have entered clinical trials, and include two gene therapy approaches. The first approach uses either the proximal hTERT (telomerase catalytic protein component) promoter to make a general cancer-specific oncolytic virus or the hTR promoter (template RNA component) to target a suicide gene therapy vector. The more advanced clinical trials include a telomerase-specific vaccine or immunotherapy; and the use of a small molecule oligonucleotide therapy that acts as a telomerase template antagonist. These approaches should not be seen as competitive, as they each target a separate aspect of telomerase cancer biology. No single trial will address all issues about how telomerase therapeutics will behave in the human body. Rather, progress in any one area will in part validate telomerase as a clinically useful target.

## CLINICAL DEVELOPMENT OF TELOMERASE THERAPEUTICS

### Gene therapy

No gene therapy product has yet been approved for the treatment of cancer in Europe or USA, and considerable technical hurdles remain to be resolved. However, there is a generally positive attitude towards the concept of gene therapy as a selective biological approach to treat cancer, and a belief within academia and in at least some industrial sectors that this is a therapeutic option that will eventually finds its place. However, this is tempered by the predictions that this mode of therapy still remains a long way from the market. More investment, development work and technical innovation are required to provide clinical validation of gene therapy, and telomerase may provide an opportunity here (Keith and Bilsland, 2008).

The concept of targeting cancer cells with a telomerase inhibitor assumes that an absence of telomerase will lead to a shortening of the telomeres at every successive cell division. Over a number of cell cycles, the chromosomes will become unstable and the cell will no longer be viable. This will lead to cell death throughout the tumour and the clinical effect will be tumour shrinkage (Keith *et al*, 2007). An alternate approach to reduce the lag time is to induce apoptotic pathways that can be coupled to telomerase activity. For example, one could hijack a central control mechanism that is responsible for regulating the expression of telomerase in the cell, and use it to drive a suicide gene or replication-competent adenovirus replication that will rapidly kill the telomerase-expressing cancer cells (Keith *et al*, 2004).

There are two general approaches to telomerase gene therapy, suicide gene therapy and oncolytic viral therapy. As a whole, the preclinical studies of telomerase gene therapy have shown a remarkable attention to specificity and efficacy using normal cells strains and an extensive array of cancer models. Both the hTERT and hTR promoters show promise in proof-of-concept studies in both the oncolytic virus and suicide gene therapy settings, with some minor concerns about off-target effects now emerging from oncolytic virus studies (Keith *et al*, 2004, 2007; Cairney and Keith, 2008).

#### Ad-hTR-NTR: a telomerase-targeted adenoviral suicide gene therapy vector

**Figure 1:**
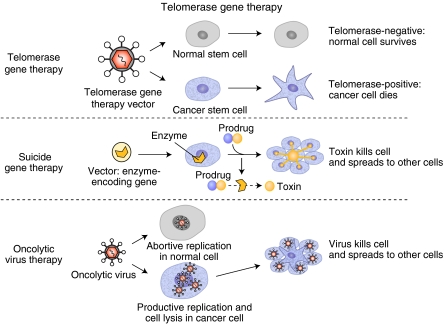
Telomerase gene therapy approaches. Antisense gene therapy such as genetic approaches to target hTERT mRNA, siRNA-mediated inhibition of a component of telomerase and so on, should selectively affect cells that are telomerase positive, while sparing telomerase-negative cells. This would be expected to take a period of time before telomerase-expressing cells died. An approach that may speed up the efficacy of gene therapy involves targeting to all cells a vector that encode an enzyme that when activated by a pro-drug will kill cells. The vector targeting telomerase is combined with an inactive enzyme and when the pro-drug is added, a toxin is released that will only kill cells that are telomerase positive. The oncolytic viral therapy takes advantage of the idea that upregulation or activation of the telomerase gene could enable the replication of a virus that could only replicate if telomerase is present. While this could theoretically affect normal stem like cells expressing telomerase, it is not clear if this will be more toxic that standard chemotherapy that affect all proliferating cells.

There are three key components in the clinical evaluation of Ad-hTR-NTR (Figure 1): adenoviral delivery of the suicide gene therapy construct; activation of the nitroreductase enzyme (NTR) by the hTR promoter and the addition of the pro-drug CB (Plumb *et al*, 2001; Bilsland *et al*, 2003). The telomerase regulation of a suicide gene therapy approach is the novel component of this therapeutic plan. The frequency of hTR expression in cancers that result in advanced intra-abdominal disease and ascites, the accessibility of these tumours both for intra-peritoneal administration of Ad-hTR-NTR and for repeat analysis of ascitic fluid for pharmacodynamic endpoints, means that these tumours are a good test model for this ‘proof-of-principle’ clinical trial of telomerase-specific suicide gene therapy. In addition, the approach of using regional administration of virus and pro-drug overcomes many of the limitations of direct intra-tumoral injection, and the intention is to use this model system as a step towards tackling the more fundamental issue of systemic administration of these viruses.

#### Clinical study:

A phase I study of Ad-hTR-NTR is due to start in 2008. This will be a single-dose, open-label, phase I study of intraperitoneal Ad-hTR-NTR in patients with advanced inoperable intra-abdominal cancer. This represents a number of different primary tumour types, defined by their location in the abdomen (including ovarian, colon, pancreatic and gastric), and also with concomitant ascites (an accumulation of fluid in the abdominal cavity). These tumour types are known to express significant levels of *hTR*, and therefore expression of the suicide gene from the *hTR* promoter should be strong. Because of the location and the presence of ascites, there is considerable opportunity for retrieving samples from the patient to monitor the progress of the therapy, and this offers an opportunity to collect valuable information that will contribute significantly to the further development of the therapeutic regimen that would not be available in other cancer models.

#### Telomerase-specific oncolytic virus

This approach utilises adenoviruses that have been manipulated or engineered to have oncolytic, or cancer-killing, properties, enabling them to selectively target and destroy cancer cells that express telomerase (Figure 1). The promoter region of the telomerase (*hTERT*) gene regulating the replication of adenovirus does permit selective viral propagation within cancer cells, but not normal cells that do not express telomerase. Thus, telomerase-expressing tumour cells containing the telomerase specific virus eventually rupture and die, spreading to adjacent cells. When these same engineered viruses infect normal somatic cells, there is no replication or killing effects. This approach is known as a Tumour-specific Replication-competent ADdenoviral (hTERTp-TRAD) gene therapy approach (Keith *et al*, 2004, 2007).

This selective lytic effect on cancer has been demonstrated *in vitro* in multiple tumour cell types, and has been extended to *in vivo* animal models (Koga *et al*, 2001; Komata *et al*, 2001; Gu *et al*, 2002). Several groups have been advancing towards clinical trials, but the work on Telomelysin, OBP-301 an attenuated adenovirus 5 vector in which the hTERT proximal promoter drives the expression of E1A and E1B genes linked with an internal ribosome entry site (Fujiwara *et al*, 2007), has recently advanced to clinical trials. This approach was developed by T Fujiwara in collaboration with Oncolys BioPharma Inc. (Minato-ku, Tokyo, Japan). In collaboration with J Nemuniatis of the Mary Crowley Medical Research Center (Dallas), the following clinical trial has recently opened: MCMRC IRB#06–12; a Phase I Dose-Escalation Study of Intratumoral Injection with Telomerase-Specific Replication-Competent Oncolytic Adenovirus, Telomelysin (OBP-301), for Various Solid Tumors (sponsor ID no. OBP-301-001). This is an open-label, dose-escalation study in which a maximum of 24 subjects will be enrolled over three dose levels. Telomelysin will be introduced intratumorally for local control in patients who have failed typical local control radiation therapy. Another company, Cell Genesys Inc., South San Francisco, CA, USA, is developing CG5757, which is engineered with a secondary telomerase promoter, employing technology licensed from Geron Corporation (Menlo Park, CA, USA), but this has not yet advanced to clinical trials.

Although this approach may be useful for local disease control, systemic treatment has the potential for side effects on telomerase competent proliferating cells. Immune cells that express telomerase are not easily infected by adenovirus. However, systemic hTERTp-TRAD might have some immediate side effects on transient amplifying stem cells such as proliferating spermatocytes in the testes, proliferating cells in the crypts of the intestine and a subset of cells in the basal and suprabasal layer of the epidermis (Wright *et al*, 1996). However, it is not certain if this approach will be any more detrimental than conventional cytotoxic drugs that affect all proliferating cells. The main problem may be that intratumoral hTERTp-TRAD injections may be limited in the ability of the adenoviral vectors to spread sufficiently and maintain tumour burden reduction for any extended periods of time.

### Telomerase (hTERT) immunotherapy

Telomerase may be a promising universal tumour antigen with broad immunotherapeutic applicability to a wide range of distinct cancers. hTERT protein is naturally processed and hTERT peptides have been shown to be presented as epitopes by the MHC, eliciting CTL responses and providing protective immunity against tumours (Minev *et al*, 2000; Nair *et al*, 2000; Vonderheide *et al*, 2001; Vonderheide, 2002). There have been two general approaches that have advanced to clinical trials.

#### Dendritic cell presentation approach

Telomerase cancer vaccine (GRNVAC1): In collaboration with the Geron Corporation, investigators at Duke University Medical Center conducted a phase I/II clinical trial on prostate cancer patients. The trial used an *ex vivo* process where dendritic cells (the most efficient antigen-presenting cells) were isolated from the patient's blood, pulsed with RNA for the telomerase protein component and then returned to the patient's body where they activated cytotoxic T cells to kill tumour cells that expressed telomerase. The trial was designed to enroll patients with metastatic prostate cancer, some who would receive three weekly vaccinations (low-dose group), while the remaining would receive six weekly vaccinations (high-dose group). Twenty patients (12 of the low-dose group and eight of the high-dose group) were enrolled and treated (Su *et al*, 2005). None of the patients in either group had treatment-related adverse effects. All but one of the patients in the low-dose group showed a significant cellular immune response specific to telomerase. All eight patients in the high-dose group showed robust cellular immune responses to telomerase, on the basis of tests assessing the generation of telomerase-specific cytotoxic CD8^+^ T-lymphocytes, as well as CD4^+^ lymphocytes. The immune responses in the high-dose group were strong as well as specific: peak responses were 1–2% of circulating CD8^+^ T cells having anti-telomerase activity. Levels of circulating cancer cells were also analysed. Ten subjects had elevated levels of circulating prostate cancer cells before vaccination. Nine of these 10 subjects had their levels reduced or cleared transiently after vaccination. Serum PSA was measured before, during and multiple times after vaccination to calculate PSA doubling time as a surrogate marker for treatment response. No significant change in PSA doubling time was observed in the low-dose group. A highly significant increase in PSA doubling time was observed in the high-dose group, suggestive of a clinical response to vaccination. Geron has now entered into an agreement with Merck & Co. Inc., Whitehouse Station, NJ, USA, for manufacturing cancer vaccines targeting telomerase by methods other than dendritic cell delivery.

#### Peptide vaccine

GemVax, a subsidiary of Pharmexa A/S Hørsholm, Denmark, has developed an injectable formulation of a promiscuous MHC class II peptide derived from the active site of telomerase (hTERT), GV1001, for the treatment of pancreatic (Bernhardt *et al*, 2006), liver and lung cancer (Brunsvig *et al*, 2006). After immunisation, the GV1001 peptide is processed and presented as MHC class I epitopes. GV1001 induces CD4^+^ and CD8^+^ T-cell immunity specific for hTERT. In phase I/II trials, vaccination with GV1001 demonstrated hTERT-specific T-cell responses with only minor associated side effects.

## GV1001: A THERAPEUTIC VACCINE FOR TREATMENT OF ADVANCED PANCREATIC CANCER

GV1001 exploits the fact that the immune system can actually recognise and react to parts of the telomerase molecule when it is presented in the right way. The GV1001 vaccine in a phase I/II clinical trial was tested in 38 evaluable patients with pancreatic cancer (Bernhardt *et al*, 2006). The patients were divided into three dose groups: 60, 300 and 1000 nmol. In the group of patients who received the medium–high dose, 75% of the patients had an immune response. The immune responses set in quickly, after 3–4 weeks, and was prolonged. The median lifetime of patients in the medium-high dose group was 8.6 months, compared with a median lifetime of approximately 5 months in patients treated with gemcitabine chemotherapy. The difference in median lifetime between immune responders and non-responders was 4.3 months. GV1001 is currently being investigated in two large phase III trials in pancreatic cancer. Together, the two trials, PrimoVax and TeloVac, aim to test GV1001 in more than 1600 patients. The PrimoVax trial is sponsored by Pharmexa and includes 520 pancreatic cancer patients from more than 80 centers in Europe, USA and Australia. The TeloVac trial is an investigator-sponsored trial designed and led by the National Cancer Research Institute in England. The trial includes over 1100 pancreatic cancer patients across UK.

## GV1001: A THERAPEUTIC VACCINE AGAINST HEPATOCELLULAR CARCINOMA

Hepatocellular carcinoma, which can arise from cirrhosis and/or infection with the hepatitis B or C viruses, causes approximately 600 000 deaths annually. Treatment options for these patients are limited. The HeptoVax trial (sponsored by Pharmexa) is ongoing in Spain, France and Germany, and has enrolled approximately 50 patients with advanced hepatocellular carcinoma. Subjects are receiving a single dose of cyclophosphamide 3 days before GV-1001 vaccination. GV-1001 is then given with GM-CSF three times in the first week, followed by one immunisation per week for 4 weeks. Booster immunisations are given once per month for a minimum of 6 months.

In summary, while vaccinations in patients with high-grade tumours have universally failed to provide a durable response, preventative immunotherapy could be a viable option in patients with surgically resectable tumour and perhaps in the future in patients with a high risk for cancer development.

### Targeting the RNA component of telomerase (telomerase template antagonists)

GRN163L is a short-chain oligonucleotide that is unique in its resistance to nuclease digestion in blood and tissues and its very high affinity and specificity for telomerase (Dikmen *et al*, 2005; Herbert *et al*, 2005; Gellert *et al*, 2006). The molecule has superior cellular and tissue penetration properties due to the chemistry and a 5′ lipid chain that facilitates entry into cells. GRN163L has antitumour effects in a wide range of haematological and solid tumour models and appears to be unique in its observed effects on putative cancer initiating (stem like) cells – the rare, chemotherapy-resistant cancer cells that are believed to cause cancer recurrence.

On the basis of broad *in vitro* and *in vivo* proof of concept for efficacy of telomerase inhibition in many major cancer types tested, good safety profile and excellent pharmacokinetics and bio-distribution, GRN163L has entered clinical trials (Geron Corporation). Initial trials as a single agent are ongoing in patients with refractory or relapsed CLL and in patients with advanced solid tumours (currently at 4.8 mg kg^−1^ per week). These early trials are designed to determine safety and maximum tolerated doses. GRN163L is a competitive substrate inhibitor with IC_50_ of 0.5–10 nM, with recovery time for 50% telomerase activity being 9 days and a long T½ beta. This has led to weekly dosing in clinical trials.

**Figure 2:**
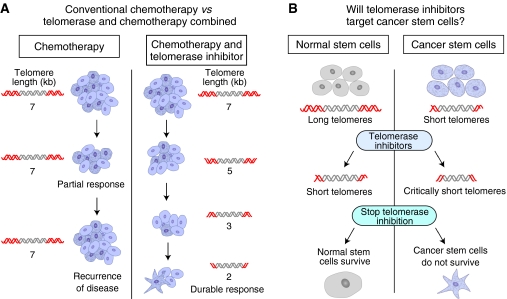
(**A**) Conventional *vs* combinatorial therapy. With standard chemotherapy, tumour burden can initially result in a partial tumour reduction response, but almost universally a subset of resistant cells results in recurrence of disease. Thus, standard chemotherapy that does not affect telomere length will results in recurrence of disease with a similar average telomere length (eg, 7 kb). In contrast, combining chemotherapy with telomerase inhibitors should results in both a partial response and a gradual shortening of telomeres (right side of figure). There is every indication on the basis of preclinical research that small oligonucleotide readily enter all cancer cells. The hope is that both sensitive and chemotherapy-resistant cells may shorten their telomeres, eventually leading to more durable responses. (**B**) Telomerase inhibitors affect stem cells and cancer cell differently. It has been reported (unpublished results) using markers of cancer stem cells that telomeres are shorter compared with normal stem cells. Thus, there should be a window of opportunity to target cancer stem cells with short telomeres using telomerase inhibitors, leading to cancer stem cell depletion before normal stem cells become critically shortened.

More recently, a GRN163L phase I trial in stage IIIB and IV non-small-cell lung cancer was initiated in combination with a standard paclitaxel/carboplatin regimen (J Schiller, University of Texas Southwestern Medical Center, Dallas). This is the first clinical trial where GRN163L is being clinically tested in combination with standard chemotherapy. While this is a phase I trial and cannot formally address issues of combination, it begins to examine the predicted mode of action of GRN163L. Preclinical data suggest that in the presence of GRN163L, a period of time will be required to shorten telomeres and thus GRN163L alone may or may not provide rapid and durable responses (Figure 2A). However combination of chemotherapy with a telomerase inhibitor should result in an initial tumour burden reduction response to chemotherapy, and over a period of weeks to months may result in progressive telomere shortening and perhaps durable responses (Figure 2A). In addition, there may be added benefits of the telomerase inhibitors if cancer-initiating (stem like) cells are also targeted. Additional trials on multiple myeloma as a single agent and in combination with velcade will initiate in the near future, and there is evidence that GRN163L may be active against myeloma stem cells (W Matsui *et al*, unpublished data).

## TELOMERASE AND CANCER STEM CELLS

Similar to normal stem cells, cancer stem (or initiating) cells also have the ability to self-renew as well as undergo differentiation to give rise to the phenotypically diverse types of cancer cells. If the hypothesis is correct and only a rare subset of tumour stem cells drives tumour formation, then the goal of cancer therapy should be to identify this population of cells and to develop therapies that target mechanisms that are more active in cancer stem cells, sparing normal tissues. In the context of this review, it is reasonable to ask if telomerase inhibitors not only target the bulk of the tumour cell population, but also the rarer cancer stem cells (Keith *et al*, 2007).

There are emerging data that suggest that normal stem cells may have longer telomeres compared with cancer stem cells (JW Shay *et al*, unpublished data), and thus there may be a window of opportunity to target cancer stem cells by inhibiting telomerase and driving telomeres short and cells into apoptotic cell death, hopefully without irreversible damage to normal stem cells (Figure 2B; Ponti *et al*, 2005; Drummond *et al*, 2007). The first and perhaps key question is if cancer stem cells even express telomerase activity. Interestingly, telomerase activity has been detected in breast cancer stem cells, with some adult tissue stem cells being telomerase negative (Ponti *et al*, 2005; Serakinci *et al*, 2006).

In addition, cancer cells cultured as spheroids on non-adherent culture dishes have many stem cell markers (eg, Oct 4, Nanog, SOX2) that are highly upregulated when compared with normal adherent cancer cell cultures. When such human spheroids are treated with GRN163L, we observe a decreased expression of these stem cell markers (JW Shay *et al*, unpublished observations). Thus, although cancer stem cells are maintained at low numbers in most solid tumours, the treatment of human solid tumours with telomerase inhibitors may shift the stem cell population from a maintenance mode into a depletion mode, eventually leading to loss of the putative stem cell population. In summary, telomerase is a universal oncology target with high tumour specificity and anti-telomerase therapies (such as GRN163L) may target the cancer stem cell population as well as the bulk of the tumour (Keith *et al*, 2007).

## CONCLUSIONS/FUTURE PERSPECTIVES

**Figure 3:**
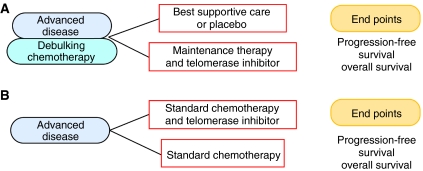
Clinical trial design for telomerase inhibitors. (**A**) A proposed phase III clinical trial design of telomerase inhibitors as maintenance therapy. This situation may be applicable to small-cell lung cancer after debulking chemotherapy. (**B**) A proposed phase III clinical trial design of telomerase inhibitors in combination with cytotoxic chemotherapy. The main endpoints are progression-free survival and overall survival.

It is encouraging to see that since the first demonstration of widespread telomerase activity in tumours 13 years ago (Kim *et al*, 1994), drug development has been rapid, with several clinical trials planned, in progress and completed. Future studies will build on this firm foundation and should hopefully demonstrate clinical value. Conventional drug development involves the assessment of toxicity and pharmacokinetics (phase I), demonstration of activity (phase II) and finally, comparison with existing standard practice (phase III). It is likely that telomerase inhibitors will have the most impact in minimal disease states such as in maintenance therapy after tumour debulking by chemotherapy or in combination with cytotoxic chemotherapy and phase III clinical trail design for these settings is possible (Figure 3). Phase III trials in advanced disease will address the questions of whether telomerase is an active target in combination or after debulking chemotherapy (Figure 3A), and if there will be an additive or synergistic effect when a telomerase inhibitor is combined with chemotherapy (Figure 3B). At the present time, it is unknown what the most appropriate ways are for combining telomerase inhibitors with established therapies. However, the emerging data from the early clinical trials will aid this stage of development. Possibilities may also exist for using telomerase inhibitors as adjuvant therapy in early disease or even chemoprevention studies. The scope for innovative clinical trial design and application is also increased through biotherapeutic approaches in immunotherapy and gene therapy.

However, although the speed at which trials have emerged is promising, there are few published biomarker studies to show that the anti-telomerase therapeutic is indeed interacting with the target as predicted. Like all mechanism-based therapeutics, telomerase inhibitors need to show clinical proof of concept. The laboratory tools, reagents and assays are available to support trial activity. Importantly, by making full use of innovative trial design, not only will the next generation of targeted therapeutics be developed, but there will also be progress in our understanding of the basic biology of human telomerase necessary to drive future waves of telomerase research.

## Figures and Tables

**Table 1:** Summary of advantages and challenges to therapeutic approaches to target telomerase cancer biology

**Approach**	**Advantages**	**Challenges**
Immunotherapy	Broadly expressed tumour-associated antigen Cell death not subject to phenotypic lag	Complex manufacture/formulation Complex biological mechanism of action Specialist clinical trial expertise required
Gene therapy	Highly tumour specific Cell death not subject to phenotypic lag	Complex manufacture/formulation Specialist clinical trial expertise required
Small-molecule oligonucleotides	Highly tumour specific Clear clinical development route	Synthetic routes for large-scale production Delayed death kill/phenotypic lag
